# Induced protein degradation for therapeutics: past, present, and future

**DOI:** 10.1172/JCI175265

**Published:** 2024-01-02

**Authors:** Hojong Yoon, Justine C. Rutter, Yen-Der Li, Benjamin L. Ebert

**Affiliations:** 1 Department of Medical Oncology, Dana-Farber Cancer Institute, Boston, Massachusetts, USA.; 2 Cancer Program, Broad Institute of MIT and Harvard, Cambridge, Massachusetts, USA.; 3 Howard Hughes Medical Institute, Boston, Massachusetts, USA.

## Abstract

The concept of induced protein degradation by small molecules has emerged as a promising therapeutic strategy that is particularly effective in targeting proteins previously considered “undruggable.” Thalidomide analogs, employed in the treatment of multiple myeloma, stand as prime examples. These compounds serve as molecular glues, redirecting the CRBN E3 ubiquitin ligase to degrade myeloma-dependency factors, IKZF1 and IKZF3. The clinical success of thalidomide analogs demonstrates the therapeutic potential of induced protein degradation. Beyond molecular glue degraders, several additional modalities to trigger protein degradation have been developed and are currently under clinical evaluation. These include heterobifunctional degraders, polymerization-induced degradation, ligand-dependent degradation of nuclear hormone receptors, disruption of protein interactions, and various other strategies. In this Review, we will provide a concise overview of various degradation modalities, their clinical applications, and potential future directions in the field of protein degradation.

## Introduction

Small molecule–induced protein degradation is a rapidly developing mode of therapeutic activity in which ubiquitin ligases are redirected to induce selective degradation of target proteins. Several degraders were used clinically before their mechanism of action was recognized (1–3), including thalidomide and its derivatives (4). Following the devastating consequences of thalidomide use during pregnancy in the 1950s that resulted in teratogenicity (5), thalidomide and its close analogs, lenalidomide and pomalidomide, were developed and approved for the treatment of multiple myeloma (6, 7) and del(5q) myelodysplastic syndrome (MDS) (8). The discovery of the mechanism of action of these degraders has opened new avenues for development of new therapeutics, including drugs that target transcription factors that have been difficult to inhibit in the past. The therapeutic potential of targeted protein degradation now encompasses a range of diverse mechanisms, including induced protein polymerization, degron exposure by disrupting protein-protein interactions, and allosteric shifts in protein structure.

**Figure 1:**
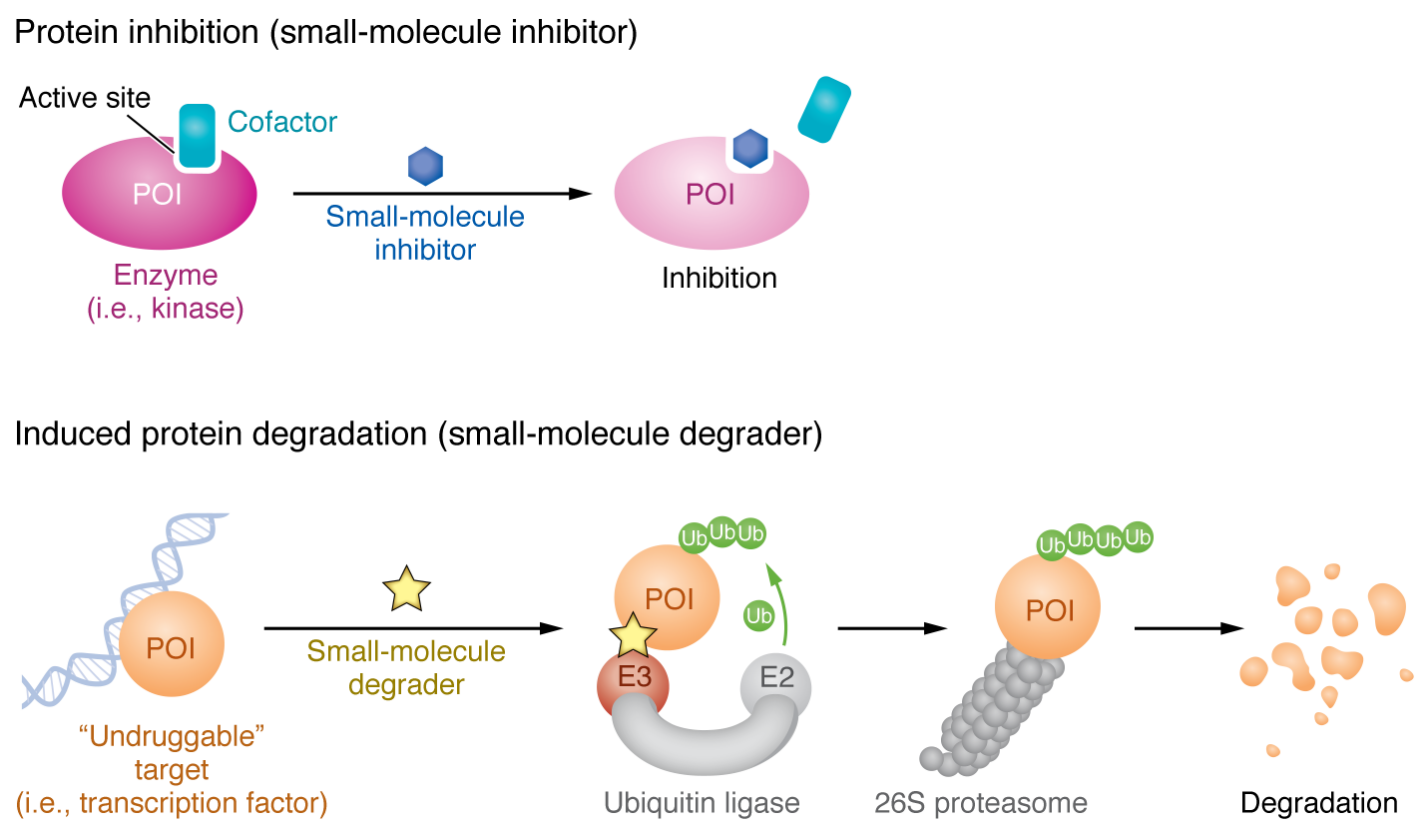
Traditional small-molecule inhibitors as compared with degraders. Traditional small-molecule inhibitors often act by blocking the catalytic function of the targeted protein of interest (POI). POIs that lack ligandable enzymatic sites have historically posed a challenge for drug discovery, earning such proteins the label of “undruggable.” Induced protein degradation, in which a small molecule facilitates the degradation of a POI, has emerged as a powerful strategy to target undruggable proteins. Degrader molecules not only expand the druggable proteome, but also enable elimination of all functions of the degraded protein, both enzymatic and scaffolding. Ub, ubiquitin.

Thalidomide analogs function as “molecular glue” degraders, small molecules that stabilize the interface between the E3 ubiquitin ligase and its “neosubstrate” protein that normally does not interact with the ligase, thereby promoting their ubiquitination and degradation in a drug-dependent manner. These analogs induce proximity between cereblon (CRBN), a substrate receptor of CRL4^^CRBN^^ ubiquitin ligase complex, and two transcription factors, IKZF1 and IKZF3 (9–11). As a result, the degradation of these master lymphoid transcription factors, which were previously considered undruggable owing to the lack of ligandable active sites, results in anticancer activity in multiple myeloma and other B cell malignancies (6, 7, 12). By circumventing the limitations of classical inhibitors and expanding the repertoire of druggable proteins, induced protein degradation holds great potential for treating a wide range of diseases (Figure 1).

Molecular glues exist in nature. The plant growth hormone auxin is a naturally occurring molecule that induces proximity between the SCF^^TIR1^^ ubiquitin ligase and Aux/IAA transcriptional repressors, leading to their degradation (13). Molecular glues with pharmacologic activity beyond degradation predate the discovery of thalidomide analogs. In the early 1990s, it was discovered that two immunosuppressants, cyclosporin A and FK506, induce protein-protein interactions between cyclophilin-calcineurin and FKBP12-calcineurin, respectively (14, 15). These interactions inhibit the phosphatase activity and immune activation function of calcineurin, effectively functioning as “molecular glue inhibitors.”

A central challenge in the field of induced protein degradation is the rational design of small-molecule degraders. To address such a challenge, heterobifunctional degraders, also known as proteolysis targeting chimeras (PROTACs) (16, 17), were developed. Heterobifunctional degraders chemically link a compound that binds a ubiquitin ligase and another that binds a target protein, which effectively recruits a ubiquitin ligase into close proximity with the target protein, leading to polyubiquitination and proteasomal degradation of the target. Many heterobifunctional degraders targeting disease-relevant proteins have been developed, and a first wave of such molecules has entered clinical trials (18–21).

Over the past decade, major strides have been made in understanding mechanisms of induced protein degradation, and many therapeutic applications are being explored. This Review provides an overview of induced protein degradation, discusses its application and challenges as a therapeutic approach, examines the various modalities of protein degradation induced by small molecules, and considers the potential implications for developing novel therapeutic strategies.

## Pharmacological manipulation of the ubiquitin-proteasome system

The majority of induced protein degradation engages the ubiquitin-proteasome system. This pathway leverages a posttranslational modification – the covalent attachment of ubiquitin to a substrate protein through the coordinated, sequential activity of three classes of enzymes (22). First, an E1 ubiquitin-activating enzyme utilizes ATP to adenylate ubiquitin and establish a thioester bond between ubiquitin and the E1 enzyme. Then, the E1 enzyme transfers the ubiquitin to an E2 ubiquitin-conjugating enzyme. Finally, an E3 ubiquitin ligase recognizes a substrate protein and facilitates the transfer of ubiquitin from the E2 enzyme to a lysine residue in the substrate protein. This ubiquitination process can be iterated, resulting in the polyubiquitination of substrate proteins, leading to a variety of cellular responses, with the most common outcome being proteasomal degradation (23). A small molecule that induces proximity between a target protein and an E3 ligase can redirect the ligase to ubiquitinate the target protein.

**Figure 2:**
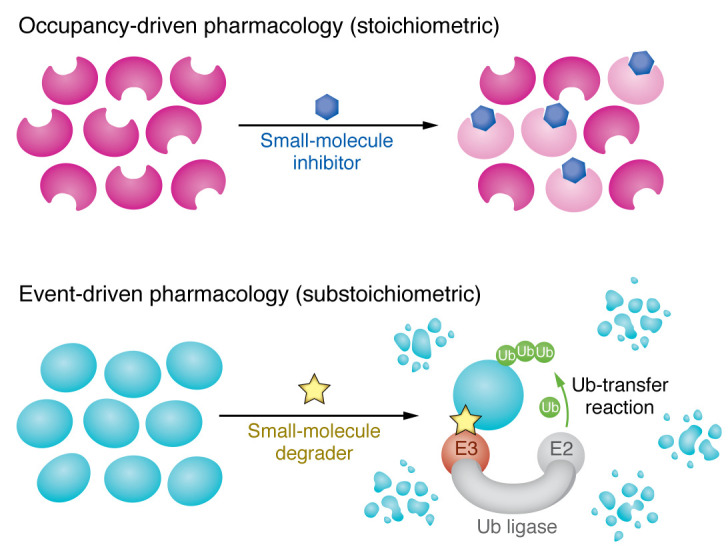
Comparison of inhibitors and degraders in pharmacology. Inhibitors rely on occupancy-driven pharmacology, which requires high drug concentrations to maintain sufficient occupancy and block the activity of proteins (occupancy-driven pharmacology).In contrast, degraders exhibit pharmacology through transient binding events that are sufficient for initiating ubiquitylation (event-driven pharmacology). These transient events can be repeated, enabling the degradation of multiple copies of the protein with substoichiometric drug concentrations. Consequently, degraders demonstrate superior pharmacology compared with traditional inhibitors. Ub, ubiquitin.

Induced protein degradation by small molecules leads to distinct pharmacologic properties compared with other drugs (Figure 2). Traditional enzyme inhibitors exhibit “occupancy-driven” pharmacology, in which therapeutic activity requires presence of the ligands at all times to inhibit the target protein. In contrast, degraders operate through “event-driven” pharmacology, in which a transient binding event is sufficient to trigger ubiquitination and subsequent degradation of the target protein (24). As a result, a single degrader molecule can facilitate repeated rounds of activity, enabling the elimination of multiple proteins per molecule (25). Following degradation, restoration of protein function requires resynthesis of the protein, which confers sustained efficacy relative to inhibitors. Biologically, degraders remove all activity of a protein, including scaffolding function, while enzyme inhibitors remove activity of the protein while the protein remains intact (26).

Degraders can leverage the protein-protein interface between a target protein and E3 ligase, enabling remarkable target specificity (27). Moreover, this protein-protein interface can enable recruitment of proteins without a high-affinity drug-binding pocket, including transcription factors, splicing factors, and various scaffolding proteins (28). Therefore, degraders substantially expand the druggable space for small-molecule drug discovery. Additionally, degraders can exhibit remarkable target specificity by requiring a substantial protein-protein interface between a target protein and a specific E3 ligase and by leveraging multiple layers of selectivity in the ubiquitin-proteasome machinery.

## Protein degradation modalities

### CRBN-based molecular glue degraders.

Thalidomide was first marketed in Germany in 1957 as the only nonbarbiturate sedative and antiemetic to treat nausea during pregnancy (29). The drug was thought to be highly safe, as rodents displayed no toxicity even after administration of very high doses (30). Only after the introduction of thalidomide into widespread clinical use in many countries was it recognized that thalidomide is a powerful teratogen. Over a four-year period, thalidomide use resulted in nearly 10,000 infants born with birth defects, including phocomelia (5), before the drug was removed from the market in 1961. Following new discoveries about the biological activity of thalidomide, thalidomide was tested in a variety of clinical settings and had efficacy for the treatment of multiple myeloma, resulting in FDA approval in 2006 (31). However, the molecular target of thalidomide was not elucidated until 2010 when the E3 ligase CRBN was identified as the primary target using thalidomide-linked affinity purification beads (11).

In 2014, it was found that lenalidomide acts as a molecular glue, redirecting CRBN to IKZF1/3, master lymphoid transcriptional factors, for degradation (9, 10). Lenalidomide also targets CK1α for degradation (32). CK1α is encoded by a gene within the common deleted region for del(5q) MDS. Its haploinsufficient expression sensitizes cells to lenalidomide therapy, providing a mechanistic basis for the therapeutic window of lenalidomide in del(5q) MDS. Notably, lenalidomide treatment has resulted in complete cytogenetic remission in approximately 50% of patients with MDS and achieving transfusion independence in around 70% of patients with del(5q) MDS patients (33, 34).

Although considerable progress has been made in understanding the therapeutic mechanism of action of thalidomide and its analogs, the cause of thalidomide syndrome remained unknown. One possibility is the presence of neosubstrates that are only expressed during specific developmental stages, and degradation of such substrates might result in the observed birth defect. To investigate this hypothesis, a quantitative mass spectrometry approach was employed, utilizing human embryonic stem cells as a model system (35). This study identified SALL4 as a novel neosubstrate that undergoes degradation by CRBN-thalidomide. SALL4 is an embryonic transcription factor known to play a crucial role in the development of fetal limbs during embryogenesis and is genetically associated with embryopathies (36). Notably, loss-of-function mutations in SALL4 are associated with human congenital birth defects, and the clinical manifestation of these mutations significantly overlaps with thalidomide-induced phocomelia. A SALL4 mouse model with these mutations also exhibited either early embryonic lethality or defects in early embryonic development and organogenesis (37). Further genetic studies in nonhuman primates or rabbits would provide valuable insights into the consequence of SALL4 degradation in thalidomide-induced embryopathies.

Each of the neosubstrates that are degraded by thalidomide analogs share a common structural motif known as a “structural degron,” which consists of a β-hairpin loop with a glycine residue located at a conserved position (38–41). Many zinc finger proteins possess this conserved β-hairpin held together by zinc-coordinating Cys__2__-His__2__ (C2H2) residues. To explore potential neosubstrates among zinc finger-containing proteins, a comprehensive genetic approach was performed to define the human zinc finger “degrome” (40). This systematic screening of the human C2H2 zinc finger proteome led to the identification of several novel zinc fingers that can be targeted by thalidomide analogs. Structural analysis of these degrons and subsequent computational docking studies revealed that a wide range of zinc fingers with varying amino acid sequences have the potential to bind the drug-CRBN interface in the presence of different thalidomide derivatives (40). One specific example is the degradation of IKZF2 by thalidomide analogs (41, 42). IKZF2 functions as a transcription factor critical for maintaining the function and stability of regulatory T cells, and its loss promotes the secretion of proinflammatory cytokines and enhances antitumor immune responses (41). IKZF2 degraders have entered clinical trials for cancer immunotherapy (42).

Notably, various nonzinc finger proteins possessing the β-hairpin structural degron can also be recruited to the CRBN surface using structurally diverse thalidomide analogs. CRBN modulators such as eragidomide (CC-90009) effectively recruit and degrade GSPT1, which functions as a translation termination factor (43, 44). In clinical trials, eragidomide had some efficacy for the treatment of relapsed or refractory acute myeloid leukemia. However, the observed toxicity has raised concerns about its benefit-to-risk profile, casting doubt on its prospects for ongoing clinical development (45). These advances in CRBN-based molecular glue degrader discovery highlight the potential of harnessing the β-hairpin loop, which is highly abundant in the human proteome, expanding the repertoire of potential CRBN neosubstrates and offering new therapeutic opportunities.

One puzzling aspect of thalidomide analogs is their lack of activity and teratogenicity in murine models (46), despite a conserved capacity of thalidomide to bind to human and mouse CRBN (47). The sequence of human CRBN divergences from the mouse sequence at specific amino acid residues on the surface of mouse CBRN that prevent recruitment of neosubstrates due to steric hindrance (32). Substitution of Ile391 in mouse CRBN with the corresponding human valine residue (I391V) enables the degradation of IKZF1/3 and CK1α, by thalidomide, lenalidomide, and pomalidomide both in vitro and in vivo (48). Degradation of GSPT1 by CC-885 requires humanization of an additional residue (V380E) (48, 49). Genetically engineered murine models have been generated, including a single amino acid germline knockin that enables degradation of IKZF1, IKZF3, and CK1α (*CRBN*^^I391V^^) and a model that enables degradation of GSPT1 as well (*CRBN*^^V380E/I391V^^) (48, 49). These humanized Crbn mouse models are of paramount importance, particularly in that many studies evaluating the toxicity of CRBN-based degraders have relied on nonhumanized Crbn mice. These models serve as ideal tools to assess both in vivo efficacy and toxicity of this class of drugs.

Laboratory and clinical studies have provided insights into resistance mechanisms to thalidomide analogs. Aberrant expression or genetic alterations in the degradation machinery, such as mutations in CRL4^^CRBN^^ components and associated cullin-ring ligase machinery, cause resistance to thalidomide analogs (50). Inactivating mutations in CRBN, including missense mutations within the drug-binding domain and surrounding surfaces, have been identified in many patients with myeloma treated with thalidomide analogs (51–53). Epigenetic inactivation of CRBN has been observed in patients with refractory multiple myeloma (54). Resistance can arise not only through mechanisms that attenuate the degradation of target proteins but also via modulation of downstream effectors and shifts in cellular dependencies. For instance, the degradation of CK1α induced by lenalidomide, which results in p53 activation, also leads to a positive selective pressure for p53-mutant hematopoietic cells, thereby causing resistance to lenalidomide (55, 56). In addition to the resistance mechanism of thalidomide analogs, potential resistance mechanisms from CRBN-based heterobifunctional degraders have been investigated using deep mutational scanning. This approach identified several potential hot spot mutations adjacent to the CRBN warhead binding site, which are also found in patients treated with thalidomide analogs (57). Interestingly, in preclinical studies with a CDK12-targeting heterobifunctional degrader, it was observed that resistance mutations can emerge on the target protein rather than in the E3 ligase. This suggests a potential mechanism through which cells might evade the target degradation (58). Preclinical models have also revealed that cancer cells may upregulate the expression of alternative substrates, thereby preventing degradation of crucial substrates involved in oncogenesis (59). Alternatively, resistance may arise through direct upregulation of target substrates. Confirmation from clinical data is required to validate these preclinical results.

### Other molecular glue degraders.

The principles of molecular glue degraders have been extended with the identification of additional E3 ligases that can be modulated with small molecules. One such example is indisulam, an aryl-sulfonamide anticancer agent, which induces degradation of an RNA splicing factor RBM39 by the CRL4^^DCAF15^^ E3 ligase (60, 61). In comparison to thalidomide analogs, indisulam demonstrates remarkable selectivity in quantitative mass spectrometry, specifically targeting only RBM39 and RBM23 for degradation (62). Further biochemical and structural analyses revealed that relatively weak affinity of indisulam to DCAF15 is still capable of inducing the formation of a DCAF15-indisulam-RBM39 complex through extensive protein-protein interfaces (62–64), which leads to highly selective RBM39 degradation. The degradation of RBM39 by indisulam results in considerable splicing defects, making hematopoietic and lymphoid cell lines particularly susceptible to indisulam treatment (65). Moreover, studies highlighted the sensitivity of neuroblastoma cell lines to RBM39 degradation by indisulam, suggesting potential clinical implications for targeting spliceosome in neuroblastoma treatment (66).

Molecular glue degraders of cyclin K facilitate interaction between an E3 ligase adaptor protein, DDB1, and CDK12. CDK12 natively binds to cyclin K to form a functional complex (67) and maintains this interaction when engaged in the drug-induced complex of DDB1-CDK12. As a result, CDK12 fills a new role as a neosubstrate receptor that presents cyclin K for ubiquitination (68–70). Structural studies revealed that the CDK inhibitor CR8 binds the active site of CDK12 and bridges the CDK12-DDB1 interface (68, 71). Importantly, the compound-induced complex mimics the overall architecture of a typical CRL4 E3 ligase complex. Here, CDK12 serves as a “neo-substrate receptor,” acting as a surrogate for DCAFs and facilitating efficient ubiquitin transfer to cyclin K and subsequently triggering its degradation. The structure highlighted that CDK12 forms extensive protein-protein interactions (~2,100 Å^^2^^) with DDB1 (68), enabling many CDK12 inhibitors, despite their distinct chemical structures, to induce the same type of CDK12-DDB1 interaction and cyclin K degradation (68–70, 72, 73). The study also demonstrated that subtle modification to the solvent-exposed moiety of a kinase inhibitor can confer molecular glue activity by engaging another E3 ligase to the target protein (74). Notably, this concept extends beyond gluing target proteins and E3 ligases, as it can be applied to other effector proteins, providing neomorphic pharmacology.

While previously described molecular glues do not form covalent bonds, covalent molecular glue degraders such as GNE11/MMH1 have been identified that induce degradation of BRD4 through interaction with DCAF16 (75). These degraders consist of a target-binding moiety, JQ1, with an appended electrophile. In the absence of BRD4, these degraders are unable to interact effectively with DCAF16. However, in the presence of BRD4, the degraders utilize BRD4 as a template to facilitate the covalent modification of the cysteine residue in DCAF16. This “template-assisted” covalent modification of DCAF16 promotes the formation of BRD4-DCAF16 complex, leading to the ubiquitination and subsequent degradation of BRD4. It is noteworthy that these covalent degraders exhibit a sustained degradation response by remaining bound to the ubiquitin ligase for an extended duration (75). The study also demonstrates that the potency and specificity of covalent molecular glue degraders can be adjusted by modulating the reactivity of the electrophilic warhead. These findings illustrate the unique properties and potential clinical benefits of covalent molecular glue degraders.

### Heterobifunctional degraders.

Conceptually, heterobifunctional degraders contain two chemical entities, one that binds target protein and another that binds an E3 ligase, as initially described in a patent filed in 1999 (76), followed by a publication in 2001 (16). Significant advances were made in 2015 with the development of high-affinity ligands for E3 ligase, such as CRBN- or VHL-based heterobifunctional degraders (18–20). It is important to note that the design of these degraders is relatively straightforward, and any nonfunctional ligand capable of binding to target proteins can be repurposed as a highly functional degrader (26). These features greatly expand the range of substrates that can be targeted through induced degradation.

Heterobifunctional degraders have several inherent challenges regarding their pharmacokinetic properties that are mainly attributed to their relatively high molecular weights (77). These challenges include poor solubility, limited cell permeability, and metabolic instability. Overcoming these issues requires significant efforts in optimizing warheads for both a target and an E3 ligase and the design of a linker. Moreover, the “hook effect” can occur when a heterobifunctional degrader reaches high concentrations, resulting in the saturation of binding to either the E3 ligase or the target. This effect leads to the formation of either a degrader-ligase or a degrader-target “binary” complex instead of the desired “ligase-degrader-target” ternary complex that promotes degradation of the target, ultimately culminating in a reduction of degradation activity (78). Consequently, careful optimization of dosing is crucial in clinical settings. Notwithstanding these challenges, extensive research has led to meaningful advancements, and heterobifunctional degraders with various therapeutic applications are now being evaluated in clinical trials (79).

The most advanced molecule in clinical trials is ARV-471, an estrogen receptor–targeting (ER-targeting) heterobifunctional degrader (80). ER degradation using selective ER degraders (SERDs) has proven clinically efficacious for the treatment of breast cancer, but incomplete degradation by SERDs often limits response (81). ARV-471 shows superior ER degradation and is also orally available (82), providing additional benefits to patients. Ongoing clinical trials are investigating its efficacy as monotherapy or in combination with CDK4/6 inhibitors for patients with metastatic breast cancer (80).

Heterobifunctional degraders can exhibit unique pharmacology by targeting multiple proteins, which leads to a synergistic effect on certain disease types. NX-2127, a BTK-targeting, CRBN-based heterobifunctional molecule, not only degrades its primary target BTK, but also concurrently downregulates IKZF1 and IKZF3 levels through CRBN ligand (83). This dual action results in T cell activation and IL-2 production, contributing an additional anticancer activity in B cell lymphomas. NX-2127 is currently being evaluated in patients with relapsed or refractory B cell malignancies (84).

As with other degraders, heterobifunctional degraders target not only the enzymatic activities of proteins, but also their scaffolding functions. For example, IRAK4 is a kinase that activates both IL-1 family receptor and Toll-like receptor inflammatory signaling (85). IRAK4 also serves as a scaffolding protein, playing a critical role in maintaining the Myd88-IRAK4-IRAK1/2 myddosome complex and facilitating downstream inflammatory signaling (86). Despite links to various indications, such as arthritis, atherosclerosis, systemic lupus erythematosus, psoriasis, and more (87), the inhibition of IRAK4 kinase activity has shown limited efficacy (88). KT-474, a heterobifunctional degrader targeting IRAK4, exhibits superior efficacy compared with IRAK4 kinase inhibitors by abolishing both the kinase and scaffolding functions of IRAK4 (89).

A degrader’s activity depends on the presence of the coopted E3 ligase. Consequently, spatial control of degrader pharmacology may be achieved by recruiting E3 ligases with tissue- or disease-specific expression. BCL-X__L,__ an antiapoptotic protein, is a well-validated cancer target with overexpression observed in several solid tumors and leukemia cells. However, inhibiting BCL-X__L__ results in on-target thrombocytopenia, which limits the clinical development of BCL-X__L__ inhibitors (90–92). By exploiting the fact that VHL is poorly expressed in platelets, DT-2216, a VHL-based heterobifunctional degrader, exhibits substantial BCL-X__L__ degradation in cancer cells while having minimal effect on platelets, rescuing the compound from on-target and dose-limiting toxicity (93). This approach also provides the potential to convert inhibitors with toxic adverse effects into selective degraders by engaging E3 ligases that are adequately expressed in the cells involved in diseases rather than in normal cells. DT2216 is currently under investigation in clinical trials for the treatment of relapsed or refractory solid tumors and hematological malignancies.

In addition to the compounds introduced above, several other heterobifunctional degraders are currently undergoing clinical evaluation, highlighting the potential of this modality in drug development.

### *Polymerization-induced degradation*.

Induced polymerization-dependent degradation offers an alternative mechanism for achieving small molecule–induced protein degradation, as exemplified by the case of BCL6. BCL6 is a master regulator of germinal center B cells and a critical oncogene in non-Hodgkin’s lymphomas (94, 95). In 2017, a series of highly structurally similar BCL6 inhibitors was developed, including BI-3802, which induced proteasomal degradation of BCL6 (96). Further investigation revealed a novel and unexpected mechanism of degradation in which the small molecule induces polymerization and subsequent ubiquitination and degradation of BCL6 (97). Upon binding of BI-3802 to the BTB domain of BCL6, the solvent-exposed hydrophobic moiety of BI-3802 induces interaction with the BTB domain of another BCL6 protein. Due to the symmetry of BCL6, BI-3802 induces bidirectional polymerization of BCL6, leading to the formation of higher-order assembly of an elongated, helical polymer of BCL6. In theory, this process could be iterated and result in the formation of an infinite polymer chain. BCL6 is an endogenous substrate of SIAH1 E3 ligase, and as polymerization occurs, SIAH1 is recruited more efficiently to the polymerized BCL6. This leads to enhanced SIAH1-dependent ubiquitination of BCL6, ultimately resulting in degradation of BCL6. Degradation of BCL6 promotes significant induction of expression of BCL6-repressed genes and antiproliferative effects BCL6-dependent lymphoma cell lines (96).

Another example of drug-induced polymerization is arsenic trioxide (ATO), which is approved for the treatment of acute promyelocytic leukemia (APL) in combination with all-trans retinoic acid (ATRA). The therapeutic efficacy of ATO is attributed to its induced degradation effect of the promyelocytic leukemia–retinoic acid receptor α (PML-RARA) oncogenic fusion protein, thereby relieving the block in myeloid differentiation of leukemic cells (98). Mechanistically, ATO binds to cysteine residues in zinc fingers of the PML protein, leading to polymerization of both PML and the PML-RARA fusion protein (99). Polymerized PML increases interaction with the small ubiquitin-like protein modifier–conjugating (SUMO-conjugating) enzyme UBC9, resulting in increased SUMOylation of PML. This SUMOylation recruits the SUMO-dependent E3 ligase RNF4 to PML, triggering its ubiquitination and subsequent degradation (100). The unique mode of action described above, wherein the degrader only interacts with the target protein and does not make any direct contact with the involved E3 ligase, results in exceptional specificity by preventing the undesired induction of neosubstrate targeting.

### Ligand-induced degradation of cognate nuclear hormone receptors.

Nuclear hormone receptors (NHRs) constitute a family of ligand-regulated transcription factors that play a crucial role in regulating a spectrum of biological processes ranging from embryonic development to cellular differentiation, reproduction, immunity, and metabolism (101–103). In response to ligands, NHRs alter transcriptional programs and, in many cases, are subsequently degraded via the ubiquitin-proteasome system (104), including thyroid hormone receptor (105), progesterone receptor (106), ER (107), glucocorticoid receptor (GR) (108), and PPARγ (109). NHRs share a modular structural organization composed of a flexible N-terminal domain, a highly conserved zinc finger DNA-binding domain, and a C-terminal ligand-binding domain whose ligand-dependent conformational state mediates binding of transcriptional coregulators (110). UBR5, a HECT-domain E3 ligase, degrades several NHRs in response to ligands through recognition of a degron within the ligand-exposed hydrophobic cleft of the C-terminal ligand-binding domain. Transcriptional coactivators, including members of the nuclear receptor coactivator (NCOA) family, bind the same ligand-exposed cleft as UBR5, thus coupling receptor activation with its subsequent degradation (111).

NHRs are widely targeted, as demonstrated by the abundance of small molecules targeting ER (112), AR (113), RARA (114), GR (115), PPARγ (116), and FXR (117, 118). SERDs include fulvestrant and elacestrant, both of which are FDA approved for the treatment of ER-positive breast cancer. The mechanism of SERD-mediated degradation depends on the chemical moieties present within the compound. SERDs with acrylic acid side chains induce UBR5-mediated degradation, while SERDs with basic amino side chains employ a distinct E3 ligase, RNF111 (111). Given the success of SERDs in clinics, there have been analogous efforts to develop selective androgen receptor degraders to address androgen antagonist resistance in prostate cancers (119). Retinoids, including ATRA, are a mainstay treatment for APL, inducing degradation of the fusion protein, PML-RARA (104). Importantly, degradation of the fusion protein is required for the therapeutic effect of retinoids in APL (120).

### Disruption of protein interactions.

Protein degradation can also be triggered by disrupting existing protein interactions with small molecules, exposing a natural degron for an E3 ligase. For example, HSP90 is a molecular chaperone protein that cooperates with HSP70 and other cochaperones to facilitate ATP-dependent refolding of a breadth of denatured client proteins. Inhibitors of HSP90 decrease the association of HSP90 with client proteins, disrupting folding, leading to proteasomal degradation (121, 122). The utility of HSP90 inhibition as a specific degrader therapeutic is limited, given the large number of HSP90 client proteins. In addition to direct HSP90 inhibition, several kinase inhibitors block recruitment of HSP90 and its cochaperone CDC37, thereby promoting misfolding and subsequent degradation (123, 124).

Small molecules that disrupt a native protein complex can promote exposure of protein surfaces with otherwise concealed degrons. For example, menin functions as a scaffolding cofactor for the histone methyltransferase MLL, which plays a critical role in the regulation of MLL-rearranged leukemia (125). Menin inhibitors disrupt the menin/MLL interaction, rendering menin susceptible to recruitment of an E3 ligase (126). Similarly, EZH2, a member of the PRC2 complex can undergo degradation upon treatment with an EZH2 inhibitor. Binding of the inhibitor to EZH2 results in its dissociation from the PRC2 complex, facilitating its recognition by CHIP and subsequent proteasomal degradation (127).

### Therapeutic targeting of endogenous induced protein degradation mechanisms.

Insights into the mechanisms governing endogenous protein degradation present opportunities for pharmacological intervention. Several cell state changes rely on the inducible activity of the ubiquitin proteasome system, such as response to hypoxia (128), oxidative stress (129), and nutrient levels (130), providing opportunities for therapeutics.

Hypoxia-inducible factor 1 α (HIF1α) is a transcription factor that plays a crucial role in cellular adaptation to low oxygen levels. Under normoxia, specific proline residues of HIF1α are hydroxylated by prolyl hydroxylase domain (PHD) enzymes, which are then recognized by the CRL2^^VHL^^ E3 ligase complex, ultimately leading to degradation of HIF1α (128). Elucidation of this mechanism enabled the development of prolyl hydroxylase inhibitors that stabilize HIF1α. This, in turn, stimulates erythropoietin synthesis and provides a therapeutic opportunity in certain forms of anemia (131).

Another inducible transcription factor, NRF2, plays a critical role in combating oxidative insults, including those arising from inflammation and metabolic toxicity. Under normal conditions, NRF2 is rapidly ubiquitinated by the CRL3^^KEAP1^^ E3 ligase, leading to swift degradation by the proteasome. KEAP1, a protein-rich in cysteine residues, is susceptible to modification and subsequent inactivation by reactive oxygen species. During oxidative stress, the inactivation of KEAP1 enhances NRF2 stability, enabling the transcriptional activation of downstream survival and adaptation programs (129, 132). There is substantial pharmacological interest in developing NRF2 activators as a protective measure against a variety of diseases where inflammation and oxidative stress play a key role in pathogenesis. Many of such compounds act as electrophiles, facilitating NRF2 activation by reacting with and inactivating KEAP1 (133). Dimethyl fumarate is a notable example that is currently employed in clinical practice to treat relapsing-remitting multiple sclerosis (134).

Prolyl hydroxylase inhibitors and KEAP1 inactivators each act to suppress degradative events induced by specific cell states, thereby stabilizing downstream substrates. In addition, there may be pharmacologic opportunities to reenforce endogenous degradation pathways by mimicking the cell state where the ligase is active.

## Concluding remarks

**Figure 3:**
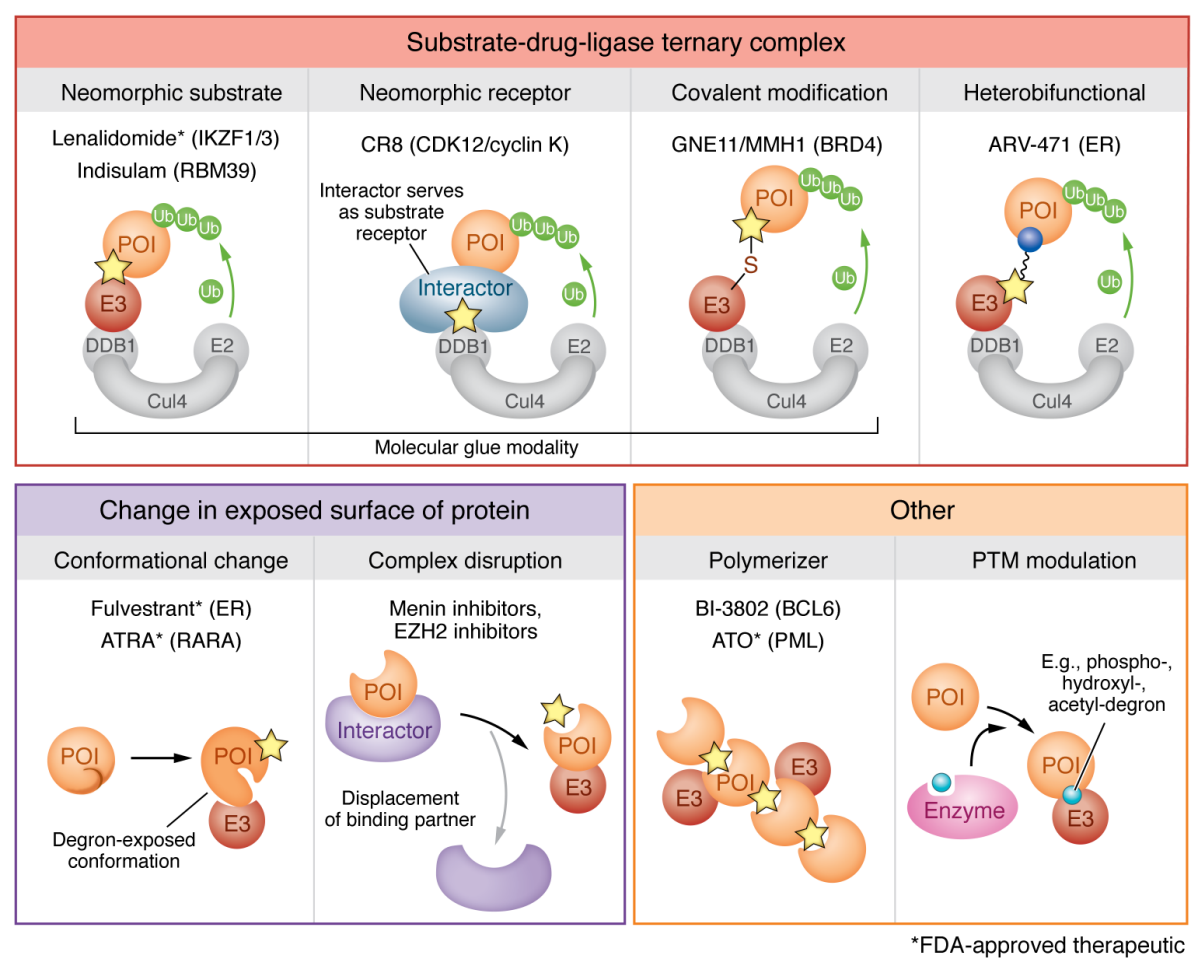
Modalities of induced protein degradation. Induced protein degradation can be achieved by several modalities, which include direct inducers of neosubstrate-ligase interactors and modulators of protein surface exposure. Molecular glues remodel protein interaction interfaces, engineering a complimentary interaction. Heterobifunctional molecules contain two binding moieties and a linker that brings two proteins into proximity. Small molecules can promote exposure of a cryptic degron by disrupting protein complexes or by inducing a conformational change. Degrons that are sensitive to posttranslational modifications (PTMs) can be modulated using small molecules that regulate the PTM state. POI, protein of interest; Ub, ubiquitin.

Recent breakthroughs in induced protein degradation have ushered in a new era in small-molecule drug discovery, unlocking pharmacologic access to targets once deemed “undruggable” and markedly improving the clinical landscape in several diseases. Small molecule–mediated modulation of degradation can be achieved through many modalities, including the direct formation of new surfaces between substrate and E3 ligase (i.e., molecular glues, heterobifunctional degraders), drug-induced changes in protein state (i.e., polymerization, degron exposure), and indirect regulation of endogenous degradation pathways (Figure 3). The potential of this new era is only just beginning to unfold, with new strategies to induce protein degradation for an expanding repertoire of recently identified and highly promising novel drug targets.

Historically, the discovery of small-molecule degraders has largely been serendipitous. However, with the conceptual framework of induced protein degradation and an improved understanding of the underlying mechanisms, the identification of small-molecule degraders is gaining momentum. This acceleration has been fueled by parallel advances in several fields, most notably in proteomics, genetic screening, biochemical assays, and structural biology. Quantitative proteomics has contributed substantially to this acceleration by enabling evaluation of the specificity of degrader molecules across the proteome, and genetic screening technologies have elucidated multiple mechanisms of induced protein degradation. Biochemical assays to evaluate inducible protein interaction, such as time-resolved fluorescence assays, can rigorously characterize small molecule–induced ternary complex formation. In addition to the progress in structural biology using cryoelectron microscopy, advances in artificial intelligence–based protein structure prediction tools could provide an opportunity to mine for degrader molecules computationally. Continued technological progress in these areas and others promises to unveil new and intriguing findings related to protein degradation, laying the groundwork for the rational design of degrader therapeutics.

Several exciting new possibilities for pharmacological intervention have emerged from the field of proximity-induced targeted protein degradation. The concept of protein degradation induced by neomorphic protein-protein interactions has inspired a broader field of induced-proximity modulators. Recently, several induced proximity-driven approaches have been reported, such as deubiquitinase-targeting chimeras (135), lysosome-targeting chimeras (136), phosphorylation-inducing small molecules (137), tricomplex inhibitors of KRas (138), and more (139).

In summary, the field of induced protein degradation has made meaningful progress, but opportunities are only beginning to be explored to harness a wider range of E3 ligases, identify new small-molecule degraders, and explore alternative mechanisms for induced protein degradation. These efforts, coupled with the advancement of induced proximity-driven pharmacology beyond degradation, offer the potential to expand the druggability of previously intractable disease-modifying protein targets.

## Version 1 01/02/2024

Electronic publication

## References

[1] Wakeling AE (1991). A potent specific pure antiestrogen with clinical potential. Cancer Res.

[2] Wu YL (2005). Structural basis for an unexpected mode of SERM-mediated ER antagonism. Mol Cell.

[3] Yoshida H (1996). Accelerated degradation of PML-retinoic acid receptor alpha (PML-RARA) oncoprotein by all-trans-retinoic acid in acute promyelocytic leukemia: possible role of the proteasome pathway. Cancer Res.

[4] Bartlett JB (2004). The evolution of thalidomide and its IMiD derivatives as anticancer agents. Nat Rev Cancer.

[5] Vargesson N (2015). Thalidomide-induced teratogenesis: history and mechanisms. Birth Defects Res C Embryo Today.

[6] Pan B, Lentzsch S (2012). The application and biology of immunomodulatory drugs (IMiDs) in cancer. Pharmacol Ther.

[7] Singhal S (1999). Antitumor activity of thalidomide in refractory multiple myeloma. N Engl J Med.

[8] Duong VH (2012). Efficacy and safety of lenalidomide in patients with myelodysplastic syndrome with chromosome 5q deletion. Ther Adv Hematol.

[9] Kronke J (2014). Lenalidomide causes selective degradation of IKZF1 and IKZF3 in multiple myeloma cells. Science.

[10] Lu G (2014). The myeloma drug lenalidomide promotes the cereblon-dependent destruction of Ikaros proteins. Science.

[11] Ito T (2010). Identification of a primary target of thalidomide teratogenicity. Science.

[12] Baz R (2010). Single agent lenalidomide in newly diagnosed multiple myeloma: a retrospective analysis. Leuk Lymphoma.

[13] Tan X (2007). Mechanism of auxin perception by the TIR1 ubiquitin ligase. Nature.

[14] Liu J (1991). Calcineurin is a common target of cyclophilin-cyclosporin A and FKBP-FK506 complexes. Cell.

[15] Schreiber SL (2021). The rise of molecular glues. Cell.

[16] Sakamoto KM (2001). PROTACs: chimeric molecules that target proteins to the Skp1-Cullin-F box complex for ubiquitination and degradation. Proc Natl Acad Sci U S A.

[17] Schapira M (2019). Targeted protein degradation: expanding the toolbox. Nat Rev Drug Discov.

[18] Winter GE (2015). Drug development. Phthalimide conjugation as a strategy for in vivo target protein degradation. Science.

[19] Lu J (2015). Hijacking the E3 ubiquitin ligase cereblon to efficiently target BRD4. Chem Biol.

[20] Lai AC (2016). Modular PROTAC design for the degradation of oncogenic BCR-ABL. Angew Chem Int Ed Engl.

[21] Bekes M (2022). PROTAC targeted protein degraders: the past is prologue. Nat Rev Drug Discov.

[22] Nalepa G (2006). Drug discovery in the ubiquitin-proteasome system. Nat Rev Drug Discov.

[23] Haglund K, Dikic I (2005). Ubiquitylation and cell signaling. EMBO J.

[24] Cromm PM, Crews CM (2017). Targeted protein degradation: from chemical biology to drug discovery. Cell Chem Biol.

[25] Lai AC, Crews CM (2017). Induced protein degradation: an emerging drug discovery paradigm. Nat Rev Drug Discov.

[26] Gechijian LN (2018). Functional TRIM24 degrader via conjugation of ineffectual bromodomain and VHL ligands. Nat Chem Biol.

[27] Huang HT (2018). A chemoproteomic approach to query the degradable kinome using a multi-kinase degrader. Cell Chem Biol.

[28] Samarasinghe KTG, Crews CM (2021). Targeted protein degradation: A promise for undruggable proteins. Cell Chem Biol.

[29] Andrews PLR (2022). Anti-emetic effects of thalidomide: Evidence, mechanism of action, and future directions. Curr Res Pharmacol Drug Discov.

[30] Somers GF (1960). Pharmacological properties of thalidomide (alpha-phthalimido glutarimide), a new sedative hypnotic drug. Br J Pharmacol Chemother.

[31] Rehman W (2011). The rise, fall and subsequent triumph of thalidomide: lessons learned in drug development. Ther Adv Hematol.

[32] Kronke J (2015). Lenalidomide induces ubiquitination and degradation of CK1α in del(5q) MDS. Nature.

[33] List A (2005). Efficacy of lenalidomide in myelodysplastic syndromes. N Engl J Med.

[34] List A (2006). Lenalidomide in the myelodysplastic syndrome with chromosome 5q deletion. N Engl J Med.

[35] Donovan KA (2018). Thalidomide promotes degradation of SALL4, a transcription factor implicated in Duane Radial Ray syndrome. Elife.

[36] Matyskiela ME (2018). SALL4 mediates teratogenicity as a thalidomide-dependent cereblon substrate. Nat Chem Biol.

[37] Warren M (2007). A Sall4 mutant mouse model useful for studying the role of Sall4 in early embryonic development and organogenesis. Genesis.

[38] Petzold G (2016). Structural basis of lenalidomide-induced CK1α degradation by the CRL4(CRBN) ubiquitin ligase. Nature.

[39] Matyskiela ME (2016). A novel cereblon modulator recruits GSPT1 to the CRL4(CRBN) ubiquitin ligase. Nature.

[40] Sievers QL (2018). Defining the human C2H2 zinc finger degrome targeted by thalidomide analogs through CRBN. Science.

[41] Wang ES (2021). Acute pharmacological degradation of Helios destabilizes regulatory T cells. Nat Chem Biol.

[42] Bonazzi S (2023). Discovery and characterization of a selective IKZF2 glue degrader for cancer immunotherapy. Cell Chem Biol.

[43] Hansen JD (2021). CC-90009: a cereblon E3 ligase modulating drug that promotes selective degradation of GSPT1 for the treatment of acute myeloid leukemia. J Med Chem.

[44] Surka C (2021). CC-90009, a novel cereblon E3 ligase modulator, targets acute myeloid leukemia blasts and leukemia stem cells. Blood.

[45] Uy GL (2019). Clinical Activity of CC-90009, a Cereblon E3 ligase modulator and first-in-class GSPT1 degrader, as a single agent in patients with relapsed or refractory acute myeloid leukemia (R/R AML): first results from a phase I dose-finding study. Blood.

[46] Fratta ID (1965). Teratogenic effects of thalidomide in rabbits, rats, hamsters, and mice. Toxicol Appl Pharmacol.

[47] Gemechu Y (2018). Humanized cereblon mice revealed two distinct therapeutic pathways of immunomodulatory drugs. Proc Natl Acad Sci U S A.

[48] Fink EC (2018). *Crbn*^I391V^ is sufficient to confer in vivo sensitivity to thalidomide and its derivatives in mice. Blood.

[49] Sellar RS (2022). Degradation of GSPT1 causes TP53-independent cell death in leukemia while sparing normal hematopoietic stem cells. J Clin Invest.

[50] Sievers QL (2018). Genome-wide screen identifies cullin-RING ligase machinery required for lenalidomide-dependent CRL4^CRBN^ activity. Blood.

[51] Kortum KM (2016). Targeted sequencing of refractory myeloma reveals a high incidence of mutations in CRBN and Ras pathway genes. Blood.

[52] Thakurta A (2014). Absence of mutations in cereblon (CRBN) and DNA damage-binding protein 1 (DDB1) genes and significance for IMiD therapy. Leukemia.

[53] Huang SY (2014). Expression of cereblon protein assessed by immunohistochemicalstaining in myeloma cells is associated with superior response of thalidomide- and lenalidomide-based treatment, but not bortezomib-based treatment, in patients with multiple myeloma. Ann Hematol.

[54] Haertle L (2021). Cereblon enhancer methylation and IMiD resistance in multiple myeloma. Blood.

[55] Sperling AS (2022). Lenalidomide promotes the development of TP53-mutated therapy-related myeloid neoplasms. Blood.

[56] Martinez-Hoyer S (2020). Loss of lenalidomide-induced megakaryocytic differentiation leads to therapy resistance in del(5q) myelodysplastic syndrome. Nat Cell Biol.

[57] Hanzl A (2023). Functional E3 ligase hotspots and resistance mechanisms to small-molecule degraders. Nat Chem Biol.

[58] Jiang B (2021). Discovery and resistance mechanism of a selective CDK12 degrader. Nat Chem Biol.

[59] Sperling AS (2019). Patterns of substrate affinity, competition, and degradation kinetics underlie biological activity of thalidomide analogs. Blood.

[60] Han T (2017). Anticancer sulfonamides target splicing by inducing RBM39 degradation via recruitment to DCAF15. Science.

[61] Uehara T (2017). Selective degradation of splicing factor CAPERα by anticancer sulfonamides. Nat Chem Biol.

[62] Faust TB (2020). Structural complementarity facilitates E7820-mediated degradation of RBM39 by DCAF15. Nat Chem Biol.

[63] Bussiere DE (2020). Structural basis of indisulam-mediated RBM39 recruitment to DCAF15 E3 ligase complex. Nat Chem Biol.

[64] Du X (2019). Structural basis and kinetic pathway of RBM39 recruitment to DCAF15 by a sulfonamide molecular glue E7820. Structure.

[65] Wang E (2019). Targeting an RNA-binding protein network in acute myeloid leukemia. Cancer Cell.

[66] Singh S (2021). Targeting the spliceosome through RBM39 degradation results in exceptional responses in high-risk neuroblastoma models. Sci Adv.

[67] Kohoutek J, Blazek D (2012). Cyclin K goes with Cdk12 and Cdk13. Cell Div.

[68] Slabicki M (2020). The CDK inhibitor CR8 acts as a molecular glue degrader that depletes cyclin K. Nature.

[69] Lv L (2020). Discovery of a molecular glue promoting CDK12-DDB1 interaction to trigger cyclin K degradation. Elife.

[70] Mayor-Ruiz C (2020). Rational discovery of molecular glue degraders via scalable chemical profiling. Nat Chem Biol.

[72] Jorda R (2022). 3,5,7-substituted pyrazolo[4,3-*d*]pyrimidine inhibitors of cyclin-dependent kinases and cyclin K degraders. J Med Chem.

[73] Sano O (2023). Novel quinazolin-4(3h)-one based Cyclin K degraders regulate alternative polyadenylation activity. Biochem Biophys Res Commun.

[74] Kozicka Z, Thoma NH (2021). Haven’t got a glue: Protein surface variation for the design of molecular glue degraders. Cell Chem Biol.

[77] Edmondson SD (2019). Proteolysis targeting chimeras (PROTACs) in ‘beyond rule-of-five’ chemical space: Recent progress and future challenges. Bioorg Med Chem Lett.

[78] Riching KM (2022). The importance of cellular degradation kinetics for understanding mechanisms in targeted protein degradation. Chem Soc Rev.

[79] Kong NR, Jones LH (2023). Clinical translation of targeted protein degraders. Clin Pharmacol Ther.

[80] Hamilton EPS (2022). ARV-471, an estrogen receptor (ER) PROTAC degrader, combined with palbociclib in advanced ER+/human epidermal growth factor receptor 2–negative (HER2-) breast cancer: Phase 1b cohort (part C) of a phase 1/2 study. J Clin Oncol.

[81] Patel R (2023). An emerging generation of endocrine therapies in breast cancer: a clinical perspective. NPJ Breast Cancer.

[82] Flanagan J (2019). Abstract P5-04-18: ARV-471, an oral estrogen receptor PROTAC degrader for breast cancer. Cancer Res.

[83] Robbins DW (2020). Nx-2127, a degrader of BTK and IMiD neosubstrates, for the treatment of B-cell malignancies. Blood.

[84] Mato AR (2022). NX-2127-001, a first-in-human trial of NX-2127, a Bruton’s Tyrosine kinase-targeted protein degrader, in patients with relapsed or refractory chronic lymphocytic leukemia and B-cell malignancies. Blood.

[85] De S (2018). Mechanism of dysfunction of human variants of the IRAK4 kinase and a role for its kinase activity in interleukin-1 receptor signaling. J Biol Chem.

[86] De Nardo D (2018). Interleukin-1 receptor-associated kinase 4 (IRAK4) plays a dual role in myddosome formation and Toll-like receptor signaling. J Biol Chem.

[87] Zarrin AA (2021). Kinase inhibition in autoimmunity and inflammation. Nat Rev Drug Discov.

[88] Kelleher J (2018). Targeted Degradation of IRAK4 Protein Via Heterobifunctional Small Molecules for Treatment of MYD88 Mutant Lymphoma. Blood.

[89] Skouras SM (2022). Selective IRAK4 degradation, not kinase inhibition, blocks TLR-activated NF-Kb and p38 Signaling leading to broad cytokine inhibition. J Immunol.

[90] Mason KD (2007). Programmed anuclear cell death delimits platelet life span. Cell.

[91] Schoenwaelder SM (2011). Bcl-xL-inhibitory BH3 mimetics can induce a transient thrombocytopathy that undermines the hemostatic function of platelets. Blood.

[92] Kaefer A (2014). Mechanism-based pharmacokinetic/pharmacodynamic meta-analysis of navitoclax (ABT-263) induced thrombocytopenia. Cancer Chemother Pharmacol.

[93] Khan S (2019). A selective BCL-X_L_ PROTAC degrader achieves safe and potent antitumor activity. Nat Med.

[94] Basso K, Dalla-Favera R (2010). BCL6: master regulator of the germinal center reaction and key oncogene in B cell lymphomagenesis. Adv Immunol.

[95] McLachlan T (2022). B-cell lymphoma 6 (BCL6): from master regulator of humoral immunity to oncogenic driver in pediatric cancers. Mol Cancer Res.

[96] Kerres N (2017). Chemically induced degradation of the oncogenic transcription factor BCL6. Cell Rep.

[97] Slabicki M (2020). Small-molecule-induced polymerization triggers degradation of BCL6. Nature.

[98] Lo-Coco F (2017). Progress and criticalities in the management of acute promyelocytic leukemia. Oncotarget.

[99] Zhang XW (2010). Arsenic trioxide controls the fate of the PML-RARalpha oncoprotein by directly binding PML. Science.

[100] Lallemand-Breitenbach V (2008). Arsenic degrades PML or PML-RARalpha through a SUMO-triggered RNF4/ubiquitin-mediated pathway. Nat Cell Biol.

[101] Dhiman VK (2018). Nuclear receptors in cancer — uncovering new and evolving roles through genomic analysis. Nat Rev Genet.

[102] Anyetei-Anum CS (2018). Thyroid hormone receptor localization in target tissues. J Endocrinol.

[103] Weikum ER (2017). Glucocorticoid receptor control of transcription: precision and plasticity via allostery. Nat Rev Mol Cell Biol.

[104] Zhu J (1999). Retinoic acid induces proteasome-dependent degradation of retinoic acid receptor alpha (RARalpha) and oncogenic RARalpha fusion proteins. Proc Natl Acad Sci U S A.

[105] Dace A (2000). Hormone binding induces rapid proteasome-mediated degradation of thyroid hormone receptors. Proc Natl Acad Sci U S A.

[106] Lange CA (2000). Phosphorylation of human progesterone receptors at serine-294 by mitogen-activated protein kinase signals their degradation by the 26S proteasome. Proc Natl Acad Sci U S A.

[107] Nawaz Z (1999). Proteasome-dependent degradation of the human estrogen receptor. Proc Natl Acad Sci U S A.

[108] Wallace AD, Cidlowski JA (2001). Proteasome-mediated glucocorticoid receptor degradation restricts transcriptional signaling by glucocorticoids. J Biol Chem.

[109] Hauser S (2000). Degradation of the peroxisome proliferator-activated receptor gamma is linked to ligand-dependent activation. J Biol Chem.

[110] Sever R, Glass CK (2013). Signaling by nuclear receptors. Cold Spring Harb Perspect Biol.

[111] Tsai JM (2023). UBR5 forms ligand-dependent complexes on chromatin to regulate nuclear hormone receptor stability. Mol Cell.

[112] Fernando TM (2023). Next-generation estrogen receptor–targeted therapeutics. Ann Rev Cancer Biol.

[113] Tan MH (2015). Androgen receptor: structure, role in prostate cancer and drug discovery. Acta Pharmacol Sin.

[114] Lo-Coco F (2013). Retinoic acid and arsenic trioxide for acute promyelocytic leukemia. N Engl J Med.

[115] Vandewalle J (2018). Therapeutic mechanisms of glucocorticoids. Trends Endocrinol Metab.

[116] Hernandez-Quiles M (2021). PPARgamma in metabolism, immunity, and cancer: unified and diverse mechanisms of action. Front Endocrinol (Lausanne).

[117] Fang S (2015). Intestinal FXR agonism promotes adipose tissue browning and reduces obesity and insulin resistance. Nat Med.

[118] Fu T (2019). FXR regulates intestinal cancer stem cell proliferation. Cell.

[119] Hwang DJ (2019). New generation of selective androgen receptor degraders: our initial design, synthesis, and biological evaluation of new compounds with enzalutamide-resistant prostate cancer activity. J Med Chem.

[120] Ablain J (2013). Uncoupling RARA transcriptional activation and degradation clarifies the bases for APL response to therapies. J Exp Med.

[121] Mimnaugh EG (1996). Polyubiquitination and proteasomal degradation of the p185c-erbB-2 receptor protein-tyrosine kinase induced by geldanamycin. J Biol Chem.

[122] Schneider C (1996). Pharmacologic shifting of a balance between protein refolding and degradation mediated by Hsp90. Proc Natl Acad Sci U S A.

[123] Jones LH (2018). Small-molecule kinase downregulators. Cell Chem Biol.

[124] Polier S (2013). ATP-competitive inhibitors block protein kinase recruitment to the Hsp90-Cdc37 system. Nat Chem Biol.

[125] Cierpicki T, Grembecka J (2014). Challenges and opportunities in targeting the menin-MLL interaction. Future Med Chem.

[126] Wu Y (2019). Disruption of the menin-MLL interaction triggers menin protein degradation via ubiquitin-proteasome pathway. Am J Cancer Res.

[127] Wang X (2017). A covalently bound inhibitor triggers EZH2 degradation through CHIP-mediated ubiquitination. EMBO J.

[128] Kaelin WG (2022). Von Hippel-Lindau disease: insights into oxygen sensing, protein degradation, and cancer. J Clin Invest.

[129] Yamamoto M (2018). The KEAP1-NRF2 system: a thiol-based sensor-effector apparatus for maintaining redox homeostasis. Physiol Rev.

[130] Nardone C (2023). A central role for regulated protein stability in the control of TFE3 and MITF by nutrients. Mol Cell.

[131] Schodel J, Ratcliffe PJ (2019). Mechanisms of hypoxia signalling: new implications for nephrology. Nat Rev Nephrol.

[132] Dayalan Naidu S, Dinkova-Kostova AT (2020). KEAP1, a cysteine-based sensor and a drug target for the prevention and treatment of chronic disease. Open Biol.

[133] Cuadrado A (2019). Therapeutic targeting of the NRF2 and KEAP1 partnership in chronic diseases. Nat Rev Drug Discov.

[134] Linker RA (2011). Fumaric acid esters exert neuroprotective effects in neuroinflammation via activation of the Nrf2 antioxidant pathway. Brain.

[135] Henning NJ (2022). Deubiquitinase-targeting chimeras for targeted protein stabilization. Nat Chem Biol.

[136] Banik SM (2020). Lysosome-targeting chimaeras for degradation of extracellular proteins. Nature.

[137] Siriwardena SU (2020). Phosphorylation-inducing chimeric small molecules. J Am Chem Soc.

[138] Schulze CJ (2023). Chemical remodeling of a cellular chaperone to target the active state of mutant KRAS. Science.

[139] Mullard A (2023). Proximity-inducing drugs get closer. Nat Rev Drug Discov.

